# Enhancement of Vindoline and Catharanthine Accumulation, Antioxidant Enzymes Activities, and Gene Expression Levels in *Catharanthus roseus* Leaves by Chitooligosaccharides Elicitation

**DOI:** 10.3390/md20030188

**Published:** 2022-03-03

**Authors:** Wenzhu Tang, Xiaoqi Liu, Yuning He, Fan Yang

**Affiliations:** School of Biological Engineering, Dalian Polytechnic University, Dalian 116034, China; tangwz@dlpu.edu.cn (W.T.); liuxiaoqifighting@gmail.com (X.L.); he20220128@gmail.com (Y.H.)

**Keywords:** chitooligosaccharides, *Catharanthus roseus*, vindoline, catharanthine, antioxidant enzymes activities, gene expression

## Abstract

*Catharanthus roseus* (L.) G. Don is a plant belonging to the genus *Catharanthus* of the *Apocynaceae* family. It contains more than one hundred alkaloids, of which some exhibit significant pharmacological activities. Chitooligosaccharides are the only basic aminooligosaccharides with positively charged cations in nature, which can regulate plant growth and antioxidant properties. In this study, the leaves of *Catharanthus roseus* were sprayed with chitooligosaccharides of different molecular weights (1 kDa, 2 kDa, 3 kDa) and different concentrations (0.01 μg/mL, 0.1 μg/mL, 1 μg/mL and 10 μg/mL). The fresh weights of its root, stem and leaf were all improved after chitooligosaccharides treatments. More importantly, the chitooligosaccharides elicitor strongly stimulated the accumulation of vindoline and catharanthine in the leaves, especially with the treatment of 0.1 μg/mL 3 kDa chitooligosaccharides, the contents of them were increased by 60.68% and 141.54%, respectively. Furthermore, as the defensive responses, antioxidant enzymes activities (catalase, glutathione reductase, ascorbate peroxidase, peroxidase and superoxide dismutase) were enhanced under chitooligosaccharides treatments. To further elucidate the underlying mechanism, qRT-PCR was used to investigate the genes expression levels of secologanin synthase (*SLS*), strictosidine synthase (*STR*), strictosidine glucosidase (*SGD*), tabersonine 16-hydroxylase (*T16H*), desacetoxyvindoline-4-hydroxylase (*D4H*), deacetylvindoline-4-*O*-acetyltransferase (*DAT*), peroxidase 1 (*PRX1*) and octadecanoid-responsive Catharanthus AP2-domain protein 3 (*ORCA3*). All the genes were significantly up-regulated after chitooligosaccharides treatments, and the transcription abundance of *ORCA3*, *SLS*, *STR*, *DAT* and *PRX1* reached a maximal level with 0.1 μg/mL 3 kDa chitooligosaccharides treatment. All these results suggest that spraying *Catharanthus roseus* leaves with chitooligosaccharides, especially 0.1 μg/mL of 3 kDa chitooligosaccharides, may effectively improve the pharmaceutical value of *Catharanthus roseus*.

## 1. Introduction

*Catharanthus roseus*, a well-known herb with great pharmaceutical value, has been recorded in folklore and traditional medical literature since 50 BC [1]. *Catharanthus roseus* can be used in the treatments of diabetes, menstrual regulators, hypertension, cancer and antigalactagogue etc. [2]. To date, more than 130 terpenoid indole alkaloids (TIAs), including catharanthine, vindoline, vincristine, vinblastine etc., have been isolated and identified from *Catharanthus roseus* [3]. Among them, vincristine and vinblastine have been used in antineoplastic drugs and cancer chemotherapies [4]. Furthermore, two precursors of them, catharanthine and vindoline, are also of great significance in the pharmacological functions of *Catharanthus roseus*. Eltayeb et al. have shown that *Catharanthus roseus* leaf extract can inhibit the invasive ability of two types of breast cancer cells by regulating matrix metalloproteinases activity [5]. According to Mariadi et al., the blood glucose levels in mice reduced quickly after being treated by nanoemulsion of *Catharanthus roseus* leaf extract [6]. At the same time, Goboza et al. reported that vindoline could lower blood sugar in rats by promoting the action of insulin [7]. In addition, Oguntibeju et al. found that vindoline can reduce diabetes and kidney diseases in rats [8]. However, most TIAs cannot be completely chemically synthesized, and they can only be obtained from plant extract at a very low yield. As a result, many investigations are carried out in order to enhance the production of important TIAs [9].

Since secondary metabolites of plants participate in inducible defense mechanisms, the synthesis of TIAs can be modulated by a variety of elicitors, such as jasmonates, salicylates, and ethylene [10]. Furthermore, it was shown that the content of alkaloids was increased after ultraviolet-B radiation [11]. Artemisinic acid also had incremental effects on the production of vindoline and vinblastine and up-regulated the expression of related genes in the TIAs pathway [12]. As a cell wall-derived elicitor, chitosan can increase the production of plant secondary metabolites [13]. According to Ali et al., chitosan could improve the growth of *Catharanthus roseus* and increase the accumulation of alkaloids under drought stress [14]. Moreover, it could increase the root weight of *Catharanthus roseus* and lead to higher alkaloid accumulation by activating the antioxidant defense system and inducing the expression of different genes [15]. However, chitosan is insoluble in water, and it should be dissolved in acetic acid to make chitosan nanoparticles [16]. As the hydrolysate of chitosan, chitooligosaccharides with degrees of polymerization (DP) <20 are soluble in water, and recent studies have shown that chitooligosaccharides could regulate plant growth and elicit plant innate immunity, too [17,18,19]. For example, chitooligosaccharides could significantly enhance the growth and photosynthesis of wheat seedlings [20]. Chitooligosaccharides could exert the strongest improving effect on the synthesis of beneficial secondary metabolites during soybean seeds germination [21,22]. However, according to Zou et al., chitohexaose and chitoheptaosehad had the most efficient effects on alleviating chilling stress to wheat seedlings, which suggested the DP was closely associated with the biological activities of chitooligosaccharides [23]. Thus, we were very interested in exploring whether chitooligosaccharides with different molecular weights could be used as an elicitor in the biosynthesis of TIAs in *Catharanthus roseus*.

In the present study, the elicitor effect of chitooligosaccharides on *Catharanthus roseus* was evaluated. The physiological properties, the concentration of vindoline and catharanthine and the antioxidant enzymes activities, as well as genes expression levels of key enzymes in the TIAs synthesis pathway of *Catharanthus roseus* were detected. To the best of our knowledge, this is the first report on the application of chitooligosaccharides as an inducer in the accumulation of alkaloids in *Catharanthus roseus*. During the growth period of *Catharanthus roseus*, foliar spraying with chitooligosaccharides can significantly stimulate the growth of *Catharanthus roseus*. Most importantly, the vindoline and catharanthine content and antioxidant enzymes activities in the leaves of *Catharanthus roseus* treated with chitooligosaccharides were considerably higher than that of the control group. In addition, the relative expression of most critical genes in the synthetic pathway of alkaloids increased with chitooligosaccharides treatments.

## 2. Results and Discussion

### 2.1. Growth and Morphological Characteristics

Under changes in external environmental conditions, the most direct manifestation of plants is the changes in their growth and morphological characteristics, in which the fresh weight and height can reflect the growth status of the plant directly [24]. In this experiment, the fresh weights of each part and the height of *Catharanthus roseus* were measured. According to the results shown in Table 1, the fresh weights of each part of *Catharanthus roseus* were positively regulated by chitooligosaccharides treatments. They were gradually increased when the concentration of chitooligosaccharides was from 0 to 0.1 μg/mL and decreased when their concentration increased continually. Among them, the 3 kDa chitooligosaccharides had the best effect. There was an obvious increase in root weight from 155.56 mg in the control group to 416.26 mg in the treatment of 0.1 μg/mL 3 kDa chitooligosaccharides, about a 2.68-fold increase. The maximum fresh weights of stem and leaf obtained in 0.1 μg/mL 3 kDa chitooligosaccharides treatment were about 3.40 and 2.36 folds compared with the control group, respectively. At the same condition, the plant height was 6.57 cm, about 1.75-fold compared with the control of 3.75 cm, which could be related to the positive impact of chitooligosaccharides and nutrient uptake due to enhanced root growth.

Agrochemicals provide a vast potential to improve plant productivity, but only a few compounds with growth-promoting activity have been described so far [25]. Chitosan and chitooligosaccharides are well documented growth stimulants and are recognized as stress signals by plant cells [26]. However, the DP of chitosan greatly affects the physicochemical properties of plants [27,28]. In the present study, chitooligosaccharides had elicitor effects on the root weight, stem weight and leaf weight, as well as plant height of *Catharanthus roseus*. The results showed that those physiological properties reached the highest values when *Catharanthus roseus* was treated with 0.1 μg/mL 3 kDa chitooligosaccharides. These results were in agreement with earlier findings, which showed that chitosan could promote plant growth and development when applied in low quantities. It had been assumed that chitooligosaccharides with different molecular weights might activate different signaling pathways, and they may act as a “metabolic enhancer” to modulate the carbon and nitrogen metabolism in plants [29].

### 2.2. Vindoline and Catharanthine Content

**Figure 1:**
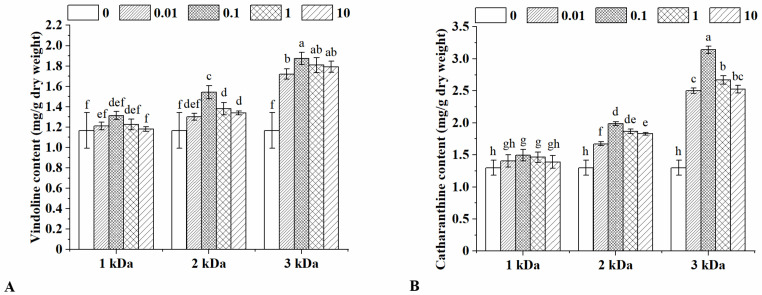
Vindoline (**A**) and catharanthine (**B**) content in the leaves of *Catharanthus roseus* under different chitooligosaccharides treatments. The treatments were conducted with chitooligosaccharides of three molecular weights (1, 2 and 3 kDa) at four different concentrations (0.01, 0.1, 1 and 10 μg/mL). Vertical bars represent the standard deviation of three replicates. Different letters indicate statistically significant difference at *p* < 0.05.

*Catharanthus roseus* is rich in alkaloids, of which vindoline and catharanthine are the key components in the synthesis of anticancer substances vinblastine and vincristine, and their high yields from the plant facilitate economical bisindole production semi-synthetically [30]. As an elicitor, chitooligosaccharides have a low molecular weight and can be recognized as stress signals by plant cells easily [31]. In the present study, the contents of vindoline and catharanthine in the leaves treated with chitooligosaccharides in different molecular weights and concentrations were measured, and the results are shown in Figure 1. The yields of vindoline and catharanthine after chitooligosaccharides treatments increased significantly. The biggest molecular weight led to the highest increase of vindoline under the same treatment concentration (Figure 1A). Vindoline content increased gradually with the increase of chitooligosaccharides until 0.1 μg/mL, and after that, it gradually reduced. With the treatment of 0.1 μg/mL 3 kDa chitooligosaccharides, the content of vindoline reached 1.88 mg/g dry weight, which was approximately 60.64% higher than that of the control. The impact of chitooligosaccharides on catharanthine also showed a similar trend (Figure 1B). The catharanthine content of *Catharanthus roseus* was increased by 15.17% with 0.1 μg/mL of 1 kDa chitooligosaccharides treatment. When the molecular weight of chitooligosaccharides increased to 3 kDa, catharanthine content reached the maximum, 3.14 mg/g dry weight, which was approximately 141.78% higher than that of the control (1.30 mg/g dry weight). Increasing the vindoline and catharanthine content due to chitooligosaccharides application may be ascribed to improved growth rate as shown in the former part, and these results were consistent with earlier findings which showed that chitosan could be used as a stimulant in alkaloids accumulation [15,32]. By comparison, chitooligosaccharides have a higher solubility than chitosan, and their use will be more convenient [33].

### 2.3. Antioxidant Enzymes Activities

The production of reactive oxygen species in *Catharanthus roseus* may increase under elicitors treatments, and a series of antioxidant enzymes are produced in order to convert the reactive oxygen species into less toxic substances and reduce oxygen damage to cell membranes, as well as improve the environment for alkaloids biosynthesis [34,35]. Therefore, it is vital to study the effects of chitooligosaccharides on the activities of various antioxidant enzymes in *Catharanthus roseus*.

**Figure 2:**
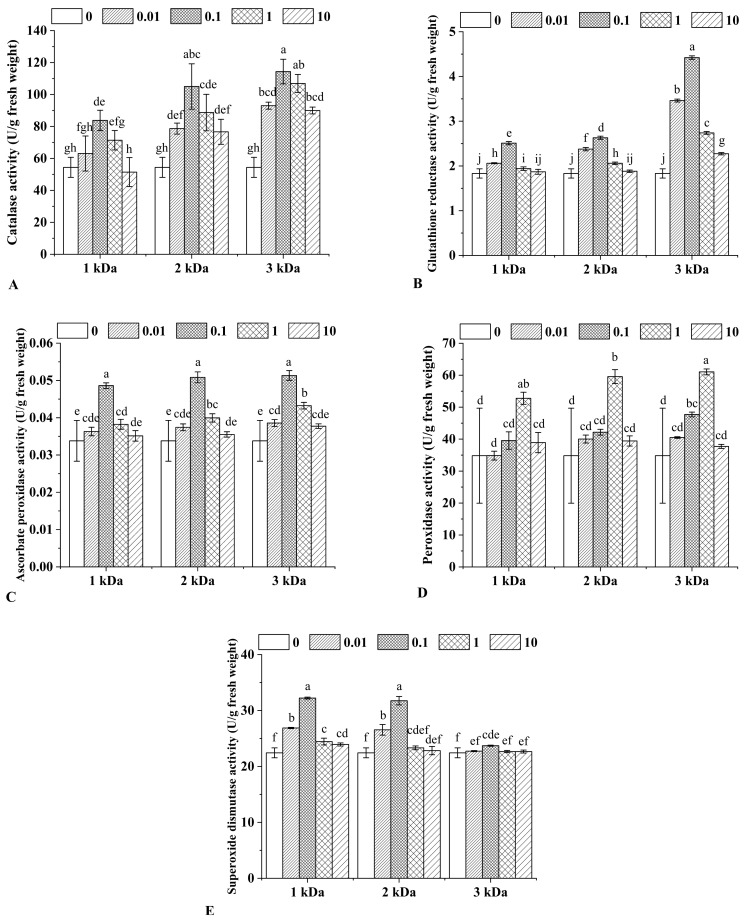
Activities of catalase (**A**), glutathione reductase (**B**), ascorbate peroxidase (**C**), peroxidase (**D**) and superoxide dismutase (**E**) in the leaves of *Catharanthus roseus* under different chitooligosaccharides treatments. The treatments were conducted with chitooligosaccharides of three molecular weights (1, 2 and 3 kDa) at four different concentrations (0.01, 0.1, 1 and 10 μg/mL). Vertical bars represent the standard deviation of three replicates. Different letters indicate statistically significant difference at *p* < 0.05.

Catalase can effectively convert excess H_2_O_2_ into water and oxygen [36]. The activity of catalase in the leaves of *Catharanthus roseus* was determined, and the results are shown in Figure 2A. Except for the 10 μg/mL 1 kDa group, chitooligosaccharides treatments enhanced the activity of catalase in *Catharanthus roseus*. With the concentration increase of chitooligosaccharides, catalase activity in leaves increased accordingly. When the concentration reached 0.1 μg/mL, catalase activity was increased to the maximum value. However, if the concentration of chitooligosaccharides was increased continually, catalase activity decreased dramatically. In the same concentration, 3 kDa chitooligosaccharides treatments had the highest promoting effects. In treatments with 0.1 μg/mL of 1 kDa, 2 kDa and 3 kDa chitooligosaccharides, catalase activity in the leaves of *Catharanthus roseus* was 83.83, 105.02 and 114.36 U/g fresh weight, respectively. Among them, 3 kDa chitooligosaccharides treatment induced the highest catalase activity, which was 110.15% higher than that of the control. It has been proved that chitooligosaccharides can improve catalase activity in barley [37]. Accordingly, it was confirmed that the spraying of chitooligosaccharides could improve the catalase activity in *Catharanthus roseus*.

Glutathione reductase is a flavoenzyme that can reduce glutathione (oxidized) (GSSH) to glutathione (reduced) (GSH), and GSH can combine with peroxides and free radicals to prevent the destruction of sulfhydryl groups and protect cells from metalion [38,39]. In the present study, glutathione reductase activity in all chitooligosaccharides treatments was significantly improved (Figure 2B), which coincided with the trend of the catalase activity. Glutathione reductase activity of *Catharanthus roseus* treated with 0.1 μg/mL of 1 kDa and 2 kDa chitooligosaccharides were 2.51 and 2.63 U/g fresh weight, which was 36.99% and 46.59% higher than that of the control. While *Catharanthus roseus* treated with 0.1 μg/mL of 3 kDa chitooligosaccharides had the highest glutathione reductase activity, which was 4.42 U/g fresh weight, representing a 141.38% increase. These results were compliant with the report of Misra et al., who clarified that saline and nitrogen sources could cause an increase of glutathione reductase and total alkaloid accumulation in the leaves of *Catharanthus roseus* [40].

Ascorbate peroxidase is an important enzyme that catalyzes H_2_O_2_-dependent ascorbate oxidation through a peroxidative one-electron transfer mechanism [41]. In the current study, *Catharanthus roseus* treated with chitooligosaccharides depicted higher activity of ascorbate peroxidase than the control (Figure 2C). Regardless of the molecular weight, ascorbate peroxidase activity first increased and then decreased with the concentration increase of chitooligosaccharides, and the best concentration was 0.1 μg/mL. The highest ascorbate peroxidase activity was 0.051 U/g fresh weight when treated by 0.1 μg/mL, 3 kDa chitooligosaccharides, increasing by 51.84% compared with the control group. These results were similar to the increase in ascorbate peroxidase activity as a result of jasmonic acid treatments [3].

Peroxidase decomposes H_2_O_2_ to produce phenolic polymers, thereby enhancing the strength of the cell wall and inhibiting the entry of heavy metals [42,43]. In the present study, the activity of peroxidase was measured to further verify the promotion effects of chitooligosaccharides on the antioxidant defense system in *Catharanthus roseus* leaves. As shown in Figure 2D, regardless of molecular weight, after chitooligosaccharides treatments, the peroxidase activity of *Catharanthus roseus* was significantly increased. When the concentration of chitooligosaccharides increased from 0 to 1 μg/mL, the activity of peroxidase gradually increased, while from 1 to 10 μg/mL, its activity decreased significantly. The peroxidase activity of *Catharanthus roseus* leaves treated with 1 μg/mL 3 kDa chitooligosaccharides showed the highest increase, reaching 75.33% more than the control. The increases of catalase and peroxidase in this experiment were similar to the result of a combination treatment with sodium nitroprusside and melatonin [44]. However, sodium nitroprusside is harmful to the environment and can cause pollution to water bodies, soil and the atmosphere. In contrast, chitooligosaccharides are more environmentally friendly and more suitable as a stimulant.

Superoxide dismutase is a front-line antioxidant enzyme catalyzing superoxide breakdown, and it is essential for most forms of eukaryotic life [45]. The effects of chitooligosaccharides on superoxide dismutase activity in *Catharanthus roseus* are illustrated in Figure 2E. For the three molecular weights, with the increased concentration of chitooligosaccharides, the activity of superoxide dismutase in *Catharanthus roseus* leaves increased, and when it was 0.1 μg/mL, it reached the maximum value. Among them, superoxide dismutase activity in *Catharanthus roseus* leaves treated with 1 kDa chitooligosaccharides was 43.66% higher than the control. The results of superoxide dismutase resembled the increase as a result of methyl jasmonate and putrescine treatments [46].

Studies have shown that oxidative stress may be important in stimulating alkaloids biosynthesis [3]. In this regard, oxidative stress occurs in treatments of chitooligosaccharides, and plants alleviated oxidative stress via increasing antioxidant enzymes activities to scavenge reactive oxygen species. The impact of chitooligosaccharides on antioxidant enzymes activities we observed was in accordance with a previous study, which reported chitooligosaccharides to have positive effects on elicitation of plant defense reactions [47]. Furthermore, it is reported that alkaloids accumulation was significantly correlated with antioxidant enzymes activities [3]. The results of this study showed that the content of vindoline and catharanthine, as well as antioxidant enzymes activities, increased as a defense response.

### 2.4. Expression Determination of Key Enzymatic Genes

Due to the complicated chemical structure of most TIAs, they can only be produced in *Catharanthus roseus* at a very low quantity [48]. In order to effectively increase the production of alkaloids in this unique plant source, the pathways of TIAs, especially the critical enzymes in the biosynthesis of alkaloids, as well as the metabolic regulation mechanism of alkaloids in *Catharanthus roseus,* have been studied in detail [49]. It is revealed that more than 35 available intermediates and 30 enzymatic steps are involved in the synthesis of *Catharanthus roseus* TIAs [50]. Overall, the secondary metabolic synthesis of alkaloids can be divided into upstream, midstream and downstream stages. In the upstream synthesis stage, strictosidine is synthesized by the coupling of tryptamine and secologanin, which are supplied by the shikimate pathway and the methylerythritol pathway/iridoid pathway, respectively. In the midstream synthesis stage, catharanthine and tabersonine are generated through a multi-step enzymatic reaction with strictosidine as the common precursor. In the downstream stage, vindoline is derived from tabersonine, and vinblastine and vincristine are synthesized by the condensation of catharanthine and vindoline [51]. These processes involve multi-step enzymatic reactions. For example, SLS and STR are engaged in the upstream stage, and SLS can catalyze the conversion of loganin to secologanin. Then, secologanin is combined with tryptamine to form strictosidine under the catalysis of STR. SGD can catalyze the removal of the glucose moiety from strictosidine to form the aglycone, which is converted to reactive dialdehyde as a precursor for the biosynthesis of TIAs. T16H, D4H and DAT are three critical enzymes in the pathways from tabersonine to vindoline, and DAT can convert deacetylvindoline to vindoline. In the downstream stage, PRX1 catalyzes the dimerisation of vindoline and catharanthine to product α-3′, 4′ anhydrovinblastine, and then vinblastine is produced in the next step. At the same time, transcriptional regulation of metabolic pathways is controlled by many transcriptional factors, and ORCA3 has been reported to participate in the regulation of secondary metabolism, and its overexpression can activate the expression of genes such as *SLS*, *STR* and *SGD*, etc. [52].

**Figure 3:**
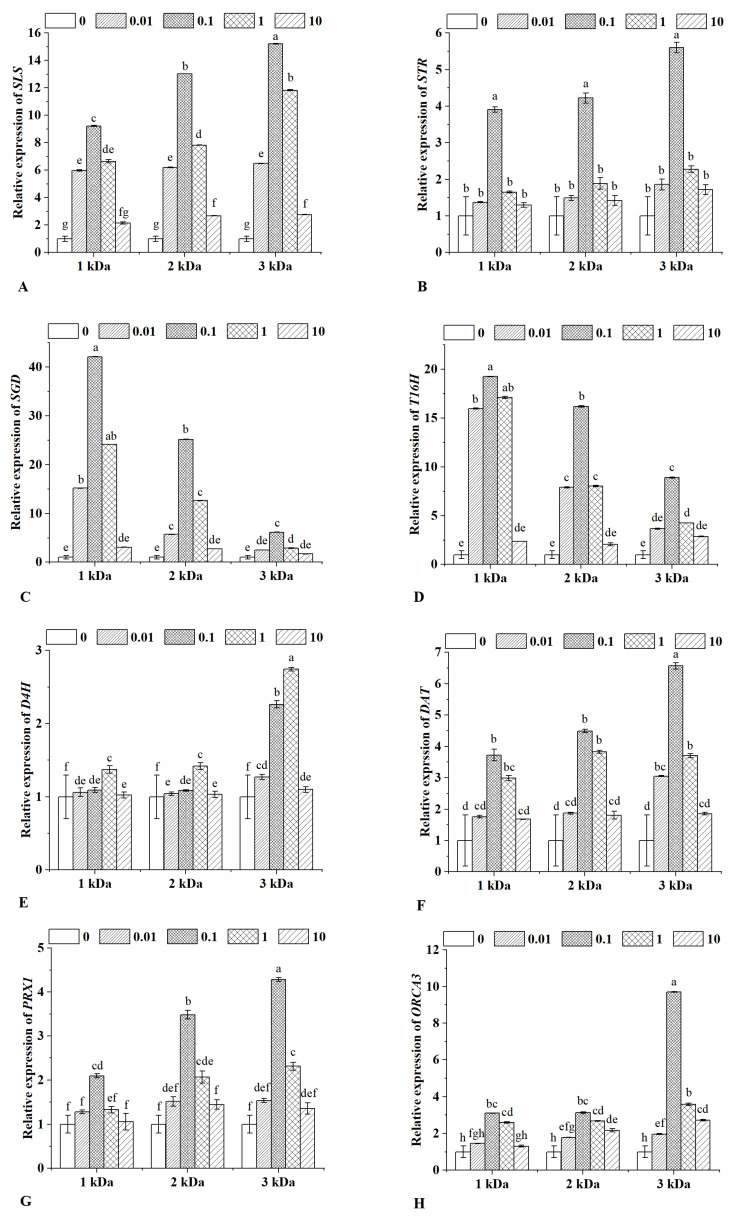
Relative expression of *SLS* (**A**), *STR* (**B**), *SGD* (**C**), *T16H* (**D**), *D4H* (**E**), *DAT* (**F**), *PRX1* (**G**) and *ORCA3* (**H**) in the leaves of *Catharanthus roseus* under different chitooligosaccharides treatments. The treatments were conducted with chitooligosaccharides of three molecular weights (1, 2 and 3 kDa) at four different concentrations (0.01, 0.1, 1 and 10 μg/mL). Vertical bars represent the standard deviation of three replicates. Different letters indicate statistically significant difference at *p* < 0.05.

In order to explore the effects of chitooligosaccharides on the synthesis of TIAs at the genetic level, qRT-PCR was used to detect the expression of the genes in the synthesis pathway of vindoline and catharanthine. As shown in Figure 3, the expressions of the *SLS*, *STR*, *SGD*, *T16H*, *D4H*, *DAT*, *PRX1* and *ORCA3* were all up-regulated with chitooligosaccharides treatments. Among them, the transcription levels of *SLS*, *STR*, *DAT*, *PRX1* and *ORCA3* significantly increased with the increase of chitooligosaccharides concentration until 0.1 μg/mL, and after that, they gradually reduced. For these five genes, the biggest molecular weight led to the highest increase of them under the same treatment concentration. Among them, the transcript abundance of *SLS*, *STR*, *DAT* and *PRX1* increased by 15.22-fold, 5.61-fold, 6.57-fold and 4.29-fold, respectively. Therefore, chitooligosaccharides might trigger the intrinsic genetic potential by promoting the transcriptional activation of key genes *SLS*, *STR*, *DAT* and *PRX1*. These results were consistent with earlier research. For example, sucrose has a positive effect on expression levels of *STR*, *DAT* and *PRX1*, as well as the production of alkaloids by activating the defense responses of plants [53]. According to Pandey et al., vindoline biosynthesis is induced by selected endophytes as a result of up-regulation of TIAs biosynthesis-related genes, including *STR*, *D4H*, *DAT* and *ORCA3* [54]. In the group of 0.1 μg/mL 3 kDa chitooligosaccharides treatment, the transcription abundance of *ORCA3* reached its maximal level, about 9.71 folds compared to the control. According to earlier research, ORCA3 plays an important role in regulating TIAs pathway genes, and its overexpression will activate the accumulation of alkaloids [53]. While after treating *Catharanthus roseus* with 1, 2 and 3 kDa chitooligosaccharides at the same concentration, the expression of *SGD* and *T16H* decreased with the increase of molecular weight.

The improvement effects reached the maximum when the molecular weight was 1 kDa, and the optimal treatment concentration was 0.1 μg/mL. The *D4H* transcription level slowly increased from 0 to 1 μg/mL of chitooligosaccharides treatments, and the highest level of transcription was observed at 1 μg/mL 3 kDa chitooligosaccharides treatment. This is similar to an earlier report, which found that the increase of vindoline coincides with the increase in *T16H* and *D4H* transcript levels after the elicitation effect of artemisinic acid [12]. In addition, the up-regulated expression of *SGD*, *D4H* and *T16H* are contributed to the increased contents of vindoline and catharanthine under binary stress of UV-B irradiation and dark incubation [55]. However, the expression of these three genes did not correlate with the change in vindoline and catharanthine accumulation in response to chitooligosaccharides treatments, suggesting that there are posttranscriptional and posttranslational mechanisms, as well as other complex regulatory mechanisms involved in a spatial and temporal manner [56]. Generally, chitooligosaccharides could stimulate the expression of the eight key genes on vindoline and catharanthine biosynthesis in the TIAs biosynthetic pathway, finally leading to the accumulation of TIAs.

## 3. Materials and Methods

### 3.1. Plant Material, Chitooligosaccharides and Chemicals

*Catharanthus roseus* seeds were preserved by the School of Biological Engineering, Dalian Polytechnic University. The soil was purchased from Shenzhibei Agricultural Technology Co., Ltd. (Baishan, China). Chitooligosaccharides with different molecular weights (1, 2 and 3 kDa) were purchased from Golden Shell Pharmaceutical Co., Ltd. (Zhejiang, China), and according to the website of the company, chitooligosaccharides products with different average molecular weights and low dispersion degrees are prepared by strictly controlled chitosan hydrolysis using specific GS chitosanase as well as membrane separation technology [57]. Vindoline and catharanthine were purchased from Yuanye Biological Technology Co., Ltd. (Shanghai, China). Methanol was purchased from Merck and Co., Inc. (Burlington, VT, USA). RNAiso Plus, PrimeScriptTM RT reagent Kit with gDNA Eraser (Perfect Real Time), TB Green Premix EX Taq II were purchased from Takara Bio Inc. (Dalian, China).

### 3.2. Treatments of Catharanthus Roseus with Chitooligosaccharides

*Catharanthus roseus* seeds were soaked in water and cultured in a constant temperature incubator (Jinghong, H-1204107, Shanghai, China). After the buds were exposed, they were transferred to the soil and cultured in a greenhouse at 25 °C with a 16 h light/8 h non-light cycle. There were 1 mL of 0.01 μg/mL, 0.1 μg/mL, 1 μg/mL and 10 μg/mL of chitooligosaccharides with different molecular weights 1 kDa, 2 kDa and 3 kDa were sprayed to the leaves at a frequency of every five days. For the control treatment, water was used instead of chitooligosaccharides. After twenty days’ treatment, three pairs of new leaves appeared, and the seedlings were collected for the next experiments.

### 3.3. Determination of the Physiological Properties

The plant height and fresh weights of root, stem and leaf of *Catharanthus roseus* were measured according to Pérez–Harguindeguy’s methods [58].

### 3.4. HPLC Analysis of Vindoline and Catharanthine Content

After harvest, the leaves in the seedlings were freeze-dried and ground into powder (Jiuyang, modelJYL-C16V, Jinan, China). Then, 100 mg of the powder was extracted with 1 mL of 95% methanol for 60 min at room temperature and centrifuged at 10,000 rpm for 15 min. The supernatant was collected and used for the vindoline and catharanthine content determination.

The content of vindoline and catharanthine was determined by HPLC with a Poroshell 120 EC-C18 column (4.6 × 250 mm, 4 µm particle size) (Waldbronn, Germany). The mobile phase consisted of water (A) and methanol (B). The flow rate was 1 mL/min, the column temperature was 30 °C, and the injection volume was 10 μL. The gradient elution process began with the mobile phase A at 80% in the first 20 min, then decreased from 80% to 20% for the next 10 min, while in the following 10 min it increased from 20% to 80%, and maintained at 80% in the last 10 min. The detection wavelengths were 310 nm and 280 nm for vindoline and catharanthine, respectively. The concentrations of vindoline and catharanthine in the sample were calculated from standard curves of each, and they were expressed as milligram per gram of dry weight.

### 3.5. Assays of Antioxidant Enzymes Activities

A crude enzyme extract was prepared by homogenizing 500 mg of leaves in 10 mL phosphate buffer (50 mM, pH 7.8) at 4 °C. The homogenates were centrifuged at 10,000 rpm for 30 min, and the supernatant was used for antioxidant enzymes activities assay.

Catalase was measured by determining the decomposition of H_2_O_2_ at 240 nm using a spectrophotometer (Molecular Devices, model SpectraMax Plus 384, San Jose, CA, USA) and ascorbate peroxidase activity was determined according to the oxidation rate of ascorbic acid at 290 nm [59]. Peroxidase activity was determined by monitoring the increase in the absorbance of 425 nm due to the oxidation of guaiacol, and superoxide dismutase was detected by following the inhibition of nitroblue tetrazolium reduction at 560 nm [59]. The activity of glutathione reductase was measured according to the increase at 412 nm due to the formation of 2-nitro-5-thiobenzoic acid [40].

### 3.6. Gene Expression Profiling and Analysis

#### 3.6.1. Primers

The genes coding the key enzymes in TIAs synthesis, as well as the reference gene, 40S ribosomal protein S9 (*RSP9*), were downloaded from the GenBank database [60], and the primers used in qRT-PCR are listed in Table 2.

#### 3.6.2. RNA Extraction and cDNA Synthesis

The leaves in the seedlings of *Catharanthus roseus* were collected and frozen in liquid nitrogen. RNAiso Plus was used to extract total RNA from the samples. The template strand cDNA was synthesized from total RNA following the instructions of PrimeScriptTM RT reagent Kit with gDNA Eraser (Perfect Real Time).

#### 3.6.3. qRT-PCR Analysis

qRT-PCR analysis was carried out for 8 TIAs biosynthesis-related genes (*SLS*, *STR*, *SGD*, *T16H*, *D4H*, *DAT*, *PRX1* and *ORCA3*). Each 25 μL reaction mixture was comprised of 2 μL cDNA (35 ng/μL), 1 μL each of PCR forward primer (10 μM) and PCR reverse primer (10 μM), and 12.5 μL TB Green Premix EX Taq II (2×) and 8.5 μL sterilized water. The PCR condition was set as 95 °C for 30 s, 40 cycles of 95 °C for 15 s and 50 °C for 20 s, and 72 °C for 15 s. The reference gene *RSP9* was used as an internal control, and the 2^−∆∆Ct^ method was used in relative quantification of the genes’ expression level [61].

### 3.7. Statistical Analysis

All tests were analyzed with at least three replicates, and data were calculated as the mean ± standard deviation. Statistical analysis was performed using SPSS software (SPSS, Chicago, IL, USA). Duncan’s multiple range test was used to compare the means at *p* < 0.05.

## 4. Conclusions

Chitooligosaccharides could significantly improve the physiological properties, alkaloids content and antioxidant properties of *Catharanthus roseus*. The root weight, stem weight, leaf weight and plant height of *Catharanthus roseus* were markedly promoted by chitooligosaccharides treatments. At the same time, the accumulation of vindoline and catharanthine was significantly increased with 0.1 μg/mL 3 kDa chitooligosaccharides treatment. Chitooligosaccharides can also improve the activities of catalase, glutathione reductase, ascorbate peroxidase, peroxidase and superoxide dismutase in *Catharanthus roseus* leaves. In addition, chitooligosaccharides mightily boosted the expression of key enzymatic genes such as *SLS*, *STR*, *SGD*, *T16H*, *D4H*, *DAT*, *PRX1* and a transcriptional regulator gene *ORCA3* in the TIAs synthesis pathway. As a result, foliar application of chitooligosaccharides could improve the accumulation of vindoline and catharanthine in *Catharanthus roseus* leaves by activating the defense responses and up-regulating the transcriptions of key genes, so chitooligosaccharides could be used as a promising abiotic elicitor for the production of TIAs.

## Figures and Tables

**Table 1:** Fresh weights of different parts (mg) and plant height (cm) of *Catharanthus roseus* under chitooligosaccharides treatments.

Treatment	COS Concentration (μg/mL)	Root Weight	Stem Weight	Leaf Weight	Plant Height
Control	0	155.56 ± 51.14 h	73.56 ± 12.58 h	297.222 ± 37.31 g	3.75 ± 0.36 f
1 kDa COS	0.01	199.84 ± 4.09 fg	84.58 ± 3.81 fgh	375.96 ± 10.60 f	4.39 ± 0.15 de
	0.1	293.30 ± 17.84 c	102.90 ± 2.83 f	434.72 ± 12.78 e	4.76 ± 0.15 cd
	1	187.38 ± 5.85 fgh	81.11 ± 4.60 gh	366.24 ± 9.49 f	4.13 ± 0.16 ef
	10	180.54 ± 13.19 fgh	75.68 ± 1.79 h	307.76 ± 6.12 g	3.99 ± 0.05 ef
2 kDa COS	0.01	255.19 ± 6.64 d	185.75 ± 15.70 d	475.73 ± 6.78 d	4.58 ± 0.07 cd
	0.1	333.91 ± 4.13 b	212.13 ± 12.10 c	557.72 ± 16.71 c	4.95 ± 0.13 c
	1	239.56 ± 4.00 de	175.44 ± 11.53 d	409.22 ± 5.13 e	4.83 ± 0.06 cd
	10	214.53 ± 3.73 ef	90.64 ± 6.38 fgh	318.81 ± 5.64 g	4.41 ± 0.16 de
3 kDa COS	0.01	332.00 ± 12.46 b	230.66 ± 4.79 b	621.57 ± 24.02 b	5.49 ± 0.37 b
	0.1	**416.26 ± 17.46 a**	**264.57 ± 10.57 a**	**702.33 ± 13.10 a**	**6.57 ± 0.16 a**
	1	300.46 ± 19.80 bc	175.44 ± 6.80 e	548.48 ± 18.34 c	5.70 ± 0.19 b
	10	172.40 ± 5.33 gh	99.16 ± 5.20 fg	355.60 ± 12.66 f	4.84 ± 0.22 cd

COS: chitooligosaccharides. Values represent mean ± standard deviation (*n* = 3). Values in a column with different letters are significantly different (*p* < 0.05). Best results in bold.

**Table 2:** Primers used for the qRT-PCR analysis.

Primer Name	Sequence (5′ → 3′)
*SLS-F*	GTTCCTTCTCACCGGAGTTG
*SLS-R*	CCCATTTGGTCAACATGTCA
*STR-F*	AAAATTCCCGATACTCCG
*STR-R*	ACCAATGGGCACTTCCTT
*SGD-F*	TCACAAAGCTGCTGTGGAAG
*SGD-R*	CACCCGTTGTTAATGGCTCT
*T16H-F*	AGGACCTTGTTGATGTGCTAC
*T16H-R*	CATTGCCCAATCGACTGTTG
*D4H-F*	TACCCTGCATGCCCTCAACC
*D4H-R*	TTGAAGGCCGCCAATTGAT
*DAT-F*	AAACCCTCTTCTCCAACCCCTC
*DAT-R*	CTTCCACGAACTCAATTCCATC
*PRX1-F*	CTGCTGCTTCTTCTCATTTCC
*PRX1-R*	CAACATCGTTTTGGAAGACCT
*ORCA3-F*	CGAATTCAATGGCGGAAAGC
*ORCA3-R*	CCTTATCTCCGCCGCGAACT
*RSP9-F*	GAGGGCCAAAACAAACTTGA
*RSP9-R*	CCCTTATGTGCCTTTGCCTA

## Data Availability

Data is contained within the article.
